# Influence of the Anisotropy on the Microstructure and Mechanical Properties of Ti/Al Laminated Composites

**DOI:** 10.3390/ma13163556

**Published:** 2020-08-12

**Authors:** Tao Huang, Zhuo Song, Fuxiao Chen, Junqing Guo, Yanbo Pei, Binghui Xing, Nan Xiang, Kexing Song

**Affiliations:** 1 School of Materials Science and Engineering, Henan University of Science and Technology, Luoyang 471023, China; huangtao@haust.edu.cn (T.H.); guojq@haust.edu.cn (J.G.); 18438581358@163.com (Y.P.); xbh1914861617@163.com (B.X.); xiangnan-87@163.com (N.X.); kxsong@haust.edu.cn (K.S.); 2 Collaborative Innovation Center of Nonferrous Metals, Luoyang 471023, China; 3 Key Laboratory of Materials science and Processing Technology for Non-ferrous Metals of Henan, Luoyang 471023, China

**Keywords:** Ti/Al laminated composites, anisotropy, microstructure, mechanical property

## Abstract

Anisotropy is the difference in the microstructure or mechanical properties of materials in different directions. Anisotropic behavior occurs in rolled sheets, and this anisotropy is very obvious in laminated composites. In this work, the influence of anisotropy on the microstructure and mechanical properties of Ti/Al laminated composites fabricated by rolling was investigated. The results show that the microstructure and mechanical properties of the Ti/Al laminated composites were obviously anisotropic. The grains in the Al layer of the composites were elongated along the rolling direction and were compressed perpendicular to the rolling direction. The grains in the Ti layer of the composites had no obvious preferential orientation and comprised mainly twins. With the rolling direction as 0°, the mechanical properties of the Ti/Al laminated composites varied greatly as the angle of the composites increased. The tensile strength, elongation and bond strength of the Ti/Al laminated composites decreased with increasing angle of the composites. In addition, the microhardness of the Ti/Al laminated composites increased with increasing angle of the composites.

## 1. Introduction

Homogenous materials may not satisfy the increasing requirements of advanced materials. Laminated composites, consisting of two or more metals, have been developed because of their improved impact behavior, corrosion, wear, bending behavior, fracture toughness and damping capacity [1,2]. A number of ways to fabricate laminated composites have been developed, such as cold rolling, hot rolling, explosive welding, and diffusion bonding. Laminated composites have excellent properties and are widely used in high-technology fields, such as aerospace and rail transit [3]. For example, Ti/Al laminated composites have the advantages of the high strength and corrosion resistance from the Ti and the light weight and high plasticity from the Al; this combination not only reduces the amount of precious metals that are required and improves the service performance of equipment, but also reduces the weight and achieves energy savings [4,5].

At ambient temperature, Ti crystals have a close-packed hexagonal structure [6,7,8], and Al crystals have a face-centered cubic structure [9]. During the rolling deformation process, grains are elongated along the rolling direction and are compressed perpendicular to the rolling direction. The grains inevitably undergo a preferred orientation to form a wire texture or a sheet texture [10,11,12]. The texture impacts the mechanical properties of materials in different directions. Materials used for practical engineering applications are generally in a biaxial stress state or a three-dimensional stress state. The mechanical properties of materials in certain directions may be weakened, which hinders the engineering application of the material. In addition, for materials used in engineering applications, it is possible to overestimate the mechanical properties of a material if the influence of a microstructural anisotropy is not considered. This may cause premature damage and fracture of the materials in their regular service life, which could result in significant safety hazards.

Due to the vast difference in the mechanical properties of Ti and Al, M. Ma et al. [13] studied the effects of rolling temperature and single-pass rolling deformation on the material strain of each layer of Ti/Al laminated composites. The results show that the ratio of the strain in the Ti and Al layers (ε_Ti_/ε_Al_) decreased as the rolling deformation increased at the same rolling temperature, and both of the ratios were less than 1. Under the same rolling deformation, the ε_Ti_/ε_Al_ increased with increasing rolling temperature, and both of the ratios were less than 1. This has important implications regarding the accurate control of the layer thickness ratio and strain ratio of each layer in Ti/Al laminated composites in actual production. Ahn et al. [14] analyzed the effect of deformation twins on the strain hardening behavior of Ti. The strain hardening rate of Ti can be split into three phases. In the initial phase, the strain hardening rate decreases with increasing strain because dislocation slip occurs readily in Ti. In the second phase, the rate increases abruptly with increasing strain due to the presence of deformation twins in Ti. In the third phase, the rate drops again with increasing strain due to dynamic recovery of Ti. D.M. Fronczek et al. [15] fabricated Ti/Al laminated composites based on explosive welding and studied the effect of annealing time on the microstructure evolution of the Ti/Al interface at an annealing temperature of 825 K. The results show that four intermediate phases were mainly formed in the bonding layer after explosion welding, namely, TiAl_3_, TiAl_2_, TiAl and Ti_3_Al. Among them, TiAl_3_ is the easiest to produce and retain. At 825 K, the other intermediate phases present at the interface were gradually converted to TiAl_3_. As the annealing continued, the content of TiAl_3_ increased continuously and spread along the entire interface, and the thickness gradually increased [15,16].

Huang et al. [16] studied the effect of annealing temperature and time on the bonding strength of a Ti/Al cladding tape. After annealing at 520 °C for 25 h, the average bonding strength of the Ti/Al cladding tape increased from 21 N/mm to 27 N/mm. Zohreh et al. [8] studied the bonding strength of Ti/Al cladding composites for different rolling amounts. The results show that when the rolling reduction rate increased to 70%, the effective bonding area and bonding strength of the Ti/Al cladding composites increased. Chen et al. [17] studied the strain models of three compounds, Ti_3_AN (A = Al, In and Tl). The elastic anisotropy of the antiperovskite Ti_3_AN was investigated, and the directionality of the atomic bonds was analyzed to reveal the nature of the elastic modulus anisotropy of the material. The results show that the anisotropy of the Ti_3_InN was significantly greater than that of the Ti_3_AlN and Ti_3_TlN, and the anisotropy was in the order of Ti_3_TlN < Ti_3_AlN < Ti_3_InN. Ning et al. [18] studied the anisotropy of the shear strength at the Zr/Ti interface and Ti/steel interface in Zr/Ti/steel laminated composites fabricated by explosive welding. The results show that the shear strength was closely related to the direction of the blast wave, namely σ at 0° > σ at 45° > σ at 90°.

Anisotropy can cause a non-uniformity in the deformation in different directions for materials used in engineering applications and increases a risk of advance damage in materials. However, there are few studies on the anisotropy of lscriptaminated composites in the existing literature [8,9,10,11,12,13,14,15,16,17,18,19,20,21]. This study focused on an in-depth analysis of the anisotropy, microstructure and mechanical properties of Ti/Al laminated composites that were fabricated by rolling in 0°, 45° and 90° directions. Morphological observation and composition analysis at the Ti/Al interface and fracture of the composites were carried out by optical microscopy (OM), scanning electron microscopy (SEM) and X-ray diffraction (XRD). The mechanical properties of the composites were studied with microhardness tests, uniaxial tensile tests and peeling tests. Upon considering the microscopic morphologies, microarea compositions and mechanical properties, the anisotropy of the Ti/Al laminated composites was determined. In this paper, the mechanism and factors that influence the anisotropy of Ti/Al laminated composites were studied to reveal the essential causes of the anisotropy. The results herein may be helpful for future research on this topic.

## 2. Materials and Methods

### 2.1. Materials

The materials used in the present study were rolled Ti/Al laminated composites. The coat plate and base plate were made from TA1 and 1060Al, respectively. The thickness of the Ti/Al laminated composites after rolling was approximately 1.90 mm, wherein the thickness of the Ti layer was 0.2 mm, the thickness of the Al layer was 1.7 mm, and the average thickness of the bonding layer was approximately 0.004 mm.

### 2.2. Metallographic Studies and Fracture Observations

To understand the microstructural variations of the Ti/Al laminated composites in different directions, metallographic specimens of the composites in the 0°, 45° and 90° directions were obtained. The Ti/Al laminated composite specimens for an examination of the microstructure were prepared by standard metallographic methods. The coat plate was etched with an etchant consisting of 2 mL of HF, 4 mL of HNO_3_, and 94 mL of H_2_O, whereas the base plate was etched with an etchant consisting of 2.5 mL of HNO_3_, 1.5 mL of HCl, 1 mL of HF, and 95 mL of H_2_O. The general microstructure characteristics of the composites were then examined under an Olympus-PMG3 type optical microscope (Olympus Corporation, Tokyo, Japan). The microstructural variations near the Ti/Al interface were analyzed under a JSM-7800F ultrahigh-resolution thermal field emission SEM (Hitachi, Tokyo, Japan). The compositional variations near the Ti/Al interface were analyzed use SEM equipped with energy-dispersive X-ray spectroscopy (EDS). The mesophase compound TiAl3 is not easily corroded. Based on this feature, the Al layer of the Ti/Al laminated composites was etched away by using a 10% volume NAOH solution. Then, the corrosive solution and corrosion products attached to the surface of the composites were washed away with an ultrasonic cleaner. The composition analysis in the bonding layer was performed on a Bruker D-8 X-ray diffractometer (Bruker AXS, Karlsruhe, Baden-Württemberg, Germany). The fractography studies of the broken specimens from the tensile tests were performed using a JSM-7800F SEM. The fracture characteristics of the Ti/Al laminated composites were determined based on the SEM observations. 

### 2.3. Mechanical Properties Testing

**Figure 1:**
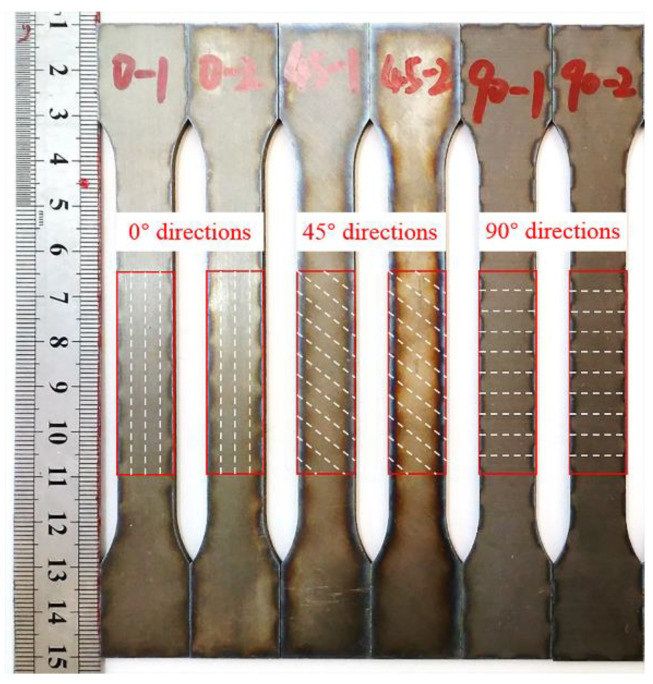
Tensile specimens.

**Figure 2:**
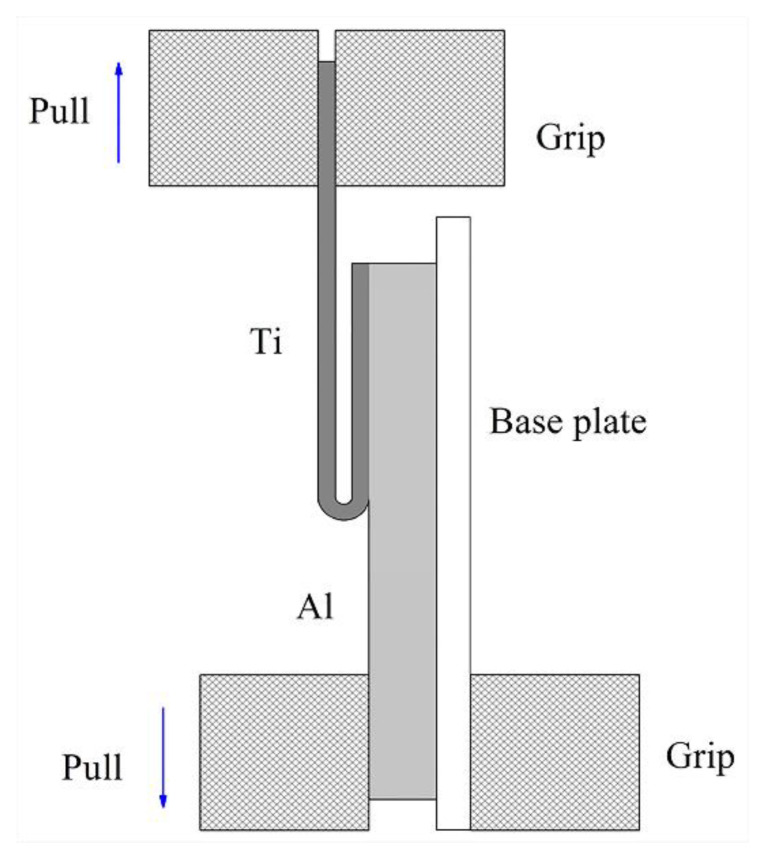
Schematic diagram of 180° peel test of the Ti/Al laminated composites.

To understand the variations in the microhardness of the Ti/Al laminated composites near the bonding interface in the different directions, microhardness testing was performed on a HVS-1000 microhardness tester (Shanghai HONC Instrument Technology Co., Ltd, Shanghai, China) for the composites in the 0°, 45° and 90° directions. The tensile specimens were designed according to national standard GB/T 228.1-2010 “Metal Material Tensile Test” [22], and the uniaxial tensile tests were carried out in a DNS100 universal electronic tensile tester. The rolling direction of the Ti/Al laminated composites was taken as the 0° direction, and three tensile specimens were taken along the 0°, 45° and 90° directions of the Ti/Al laminated composites. The tensile specimens of the Ti/Al laminated composites are shown in Figure 1 and Figure 2 show a schematic diagram of the 180° peel test. The peel specimens were fabricated with a size of 100 mm × 10 mm × 1.9 mm from the rolled Ti/Al laminated composites in the 0°, 45° and 90° directions. The peel test was carried out on a SHMADZU AG-I250KN universal electronic tensile test machine (Shimadzu, Kyoto, Japan). The peeling speed was set to 10 mm/min.

## 3. Results and Discussion

### 3.1. Microstructure and Composition Analyses

#### 3.1.1. Optical Microscopy Analysis of the Microstructure

**Figure 3:**
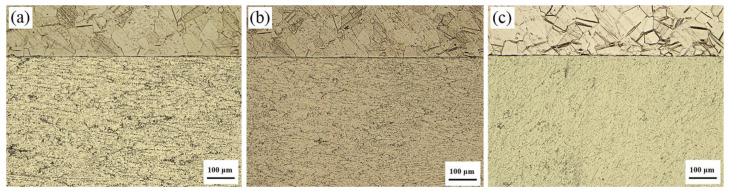
Optical microscope images of the interface in the Ti/Al laminated composites along (**a**) the 0° direction, (**b**) the 45° direction, (**c**) the 90° direction.

Figure 3 shows the results of the optical microscopy analysis of the microstructure near the Ti/Al interface in the cross-section of the Ti/Al laminated composites in the 0°, 45° and 90° directions. It can be clearly seen from Figure 3 that the Ti/Al interface was approximately straight. The crystal grains on both sides of the interface were relatively uniform, and the bonding interface was in good condition.

**Figure 4:**
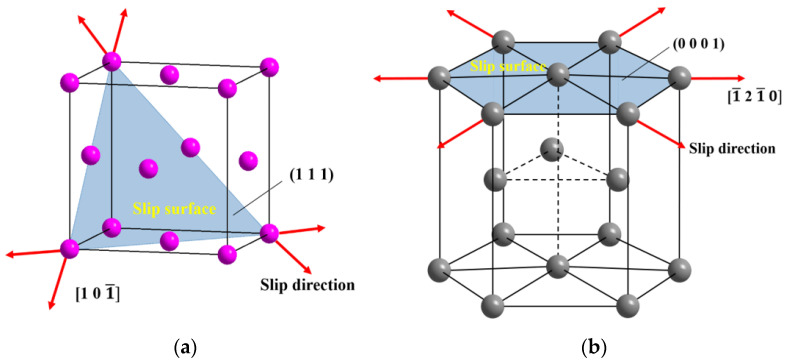
Lattice slip system of (**a**) Al and (**b**) Ti.

By comparing the Al layers in the Ti/Al laminated composites in the three directions, it can be found that the grains along the 0° direction were obviously elongated, and the grains along the 45° and 90° directions were almost regularly arranged. This may be because Al is a face-centered cubic crystal with three slip directions in each close-packed planar surface in its crystal lattice, forming a total of 12 slip systems. A large number of slip systems increase the plastic deformation ability of Al. At the same time, due to the low hardness of Al, the strength and rigidity are also relatively low, and a large deformation can be generated under a low stress. Therefore, in the rolling deformation process of the Ti/Al laminated composites, a strong rolling force caused the crystal grains in the Al materials to have a preferred orientation along the rolling direction. The grains along the rolling direction were elongated, and the grains perpendicular to the direction were compressed. The grain arrangement exhibited a significant anisotropy [11,12]. Figure 4a shows a schematic diagram of an Al lattice slip system.

By comparing the Ti layers in the Ti/Al laminated composites in the three directions, it can be seen that the grains of the Ti layers were equal in cross-sections from the three directions, and the arrangement of the grains was relatively uniform did not have an obvious anisotropy. A large number of twins were produced in the Ti layers. The closer to the interface area, the more pronounced the phenomenon of twinning was, which was also reported in other studies [23,24,25]. Ti is a hexagonal close-packed crystal with only three slip systems in its crystal lattice. Therefore, the plastic deformation ability of Ti is weak, and dislocation slip does not occur readily [26,27]. During the rolling deformation process of the Ti/Al laminated composites, the tangential deformation force generated in the Ti layers exceeded the critical shear stress for twinning due to the large deformation force. Thus, in addition to the slippage of the Ti layers, there were also a large number of twins. Figure 4b, shows a schematic diagram of a Ti lattice slip system.

In summary, the Ti/Al laminated composites fabricated by rolling had a flat interface and good bonding. Since the plasticity of the Al layers was superior to that of the Ti layers, the deformation of the Ti layers during rolling was relatively small. The Al layers had good plasticity and the Al metal flow was larger than that of the Ti layers; thus, dislocation motion and an accumulation of local dislocations occurred in the Al. Therefore, a wavy morphology was generated to a small degree at the Ti/Al bonding interface.

#### 3.1.2. Scanning Electron Microscopy (SEM) Observations and Composition Analysis at the Ti/Al Interface

**Figure 5:**
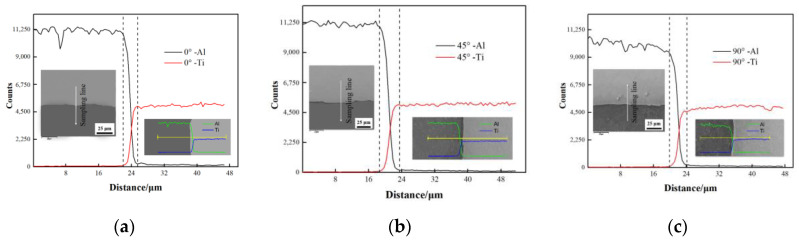
The back-scattered electron (BSE) image and micro-area element distribution across the interface of the Ti/Al laminated composites in (**a**) the 0° direction, (**b**) the 45° direction, (**c**) the 90° direction.

Figure 5 shows the microstructure of the Ti/Al interface in the Ti/Al laminated composites in different directions. It can be seen that the Ti/Al interface was basically a clean and a crack-free bright white band, with a small amount of waviness, and there was basically no difference in the bonding interfaces in the different directions. This is consistent with the analysis above.

Additionally, Figure 5 shows the back-scattered electron (BSE) image and the microelement element distribution across the Ti/Al interface. The difference in the degree of shading in a BSE image is also referred to as the contrast, which largely depends on the strength of the detected electron beam. The atomic number of the material being irradiated is one of the main factors that determines the contrast of a BSE image. During the imaging process, additional electrons are released and can be detected in materials with high atomic numbers, so the bright areas in the image indicate regions with high atomic number elements. In contrast, the light elements with low atomic numbers appear as dark areas in the image. Therefore, the element in the light-colored areas in the image is Ti, and in the dark-colored areas, it is Al. A bright white band, which is distinct from the areas with Ti and Al, was present on the Ti/Al interface. Therefore, it is speculated that new substances were formed in the bonding layer. In accordance with the existing literature [15], there were four kinds of intermediate phases in the bonding layer of the Ti/Al laminated composites, namely, TiAl_3_, TiAl_2_, TiAl and Ti_3_Al. Among them, TiAl_3_ is easiest to produce and retain. In accordance with the TiAl binary phase diagram and considering the fabrication conditions of the Ti/Al laminated composites in this paper, it is speculated that the new material in the bonding layer of the Ti/Al laminated composites prepared in this paper was TiAl_3_.

In accordance with the counts-per-second (CPS) intensity curve, the thickness of the bonding layer of the Ti/Al laminated composites was approximately 3 μm in the 0° direction, approximately 4 μm in the 45° direction, and approximately 4 μm in the 90° direction. In accordance with the electron backscattered diffraction (EBSD) pole figure of the intermetallic compound TiAl_3_, the materials in the bonding layer of the Ti/Al laminated composites had a preferred orientation [15]. Therefore, there are substantial differences in the material properties and thickness of the bonding layer in the Ti/Al laminated composites in the different directions. For the Ti/Al laminated composites fabricated by rolling in this paper, an increased bonding layer thickness means that the content of the mesophase compound TiAl_3_ near the Ti/Al interface increased. TiAl_3_ is a hard and brittle intermetallic compound, and the accumulation of TiAl_3_ at the Ti/Al interface caused a degree of distortion and hardness increase at the bonding layer of the Ti/Al laminated composites [28]. During the tensile deformation process of the Ti/Al laminated composites, the hardness distortion at the bonding layer caused a large stress concentration at the Ti/Al interface, so the cracks were generated first in the bonding layer, and a layered behavior occurred in the composites. Then, the crack gradually expanded to a Ti layer with a low ductility, followed by an Al layer, which ultimately caused the failure and destruction of the Ti/Al laminated composites [18,19,20].

**Figure 6:**
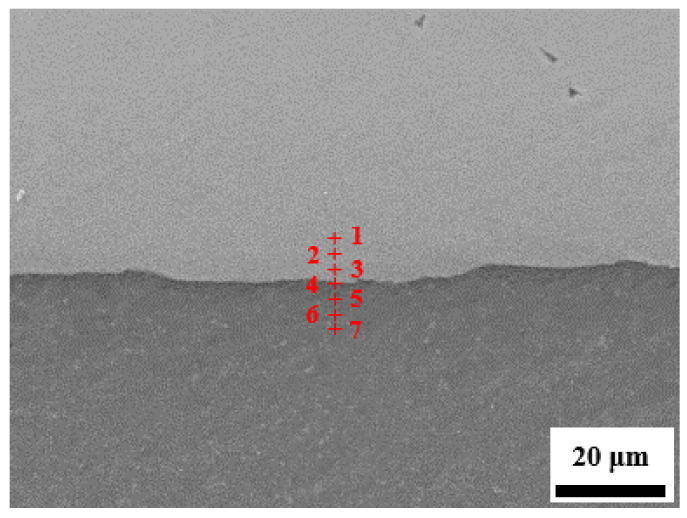
Energy-dispersive X-ray spectroscopy (EDS) analysis in bonding layer of the Ti/Al laminated composites in 45° direction.

Figure 6 shows the EDS element analysis results at the Ti/Al interface position in the Ti/Al laminated composites in the 45° direction, and the results are shown in Table 1. In accordance with the data in Table 1, it can be initially concluded that the new substance produced in the bonding layer of the Ti/Al laminated composites in this paper was TiAl_3_.

**Figure 7:**
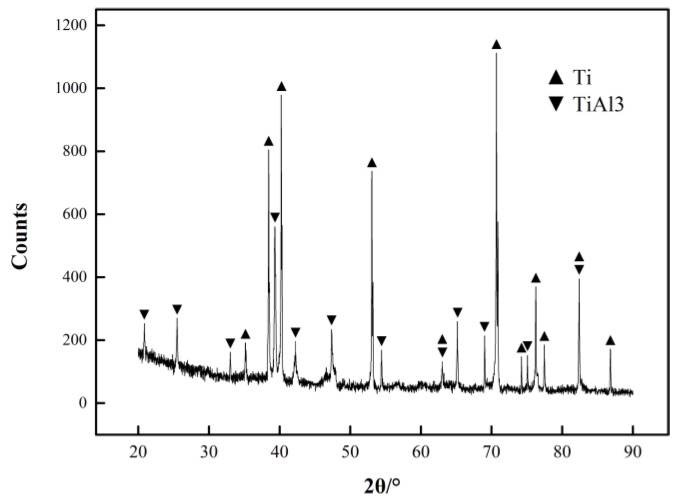
Results of the X-ray diffraction (XRD) test in the bonding layer of the Ti/Al laminated composites.

The XRD results are shown in Figure 7. In accordance with the calibration results of the object image, the new material produced in the bonding layer of the Ti/Al laminated composites in this paper was TiAl_3_ [29].

### 3.2. Mechanical Properties

#### 3.2.1. Microhardness

**Figure 8:**
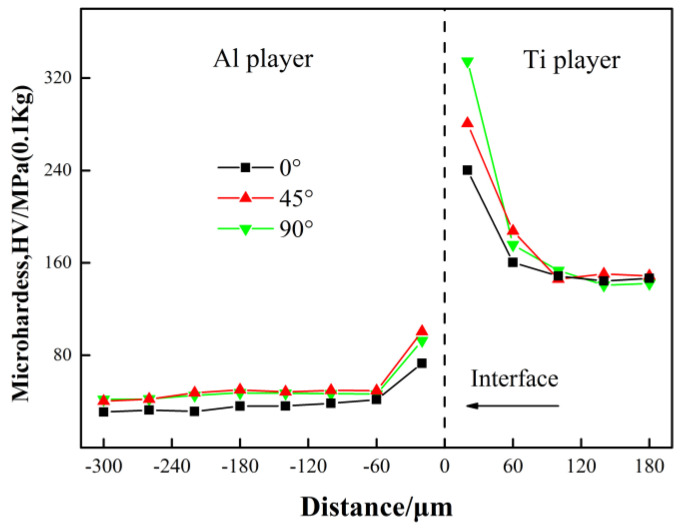
Micro-hardness near Ti/Al interface of the Ti/Al laminated composites.

Figure 8 shows the results of the Vickers hardness test near the Ti/Al interface of the Ti/Al laminated composites. As shown in Figure 8, the average hardness of the Ti layer away from the Ti/Al interface was approximately 148 HV and that of the Al layer was approximately 42 HV. The microhardness had a peak near the interface position of the Ti/Al laminated composites. This result is also consistent with studies done elsewhere [30]. In accordance with the discussion above, the hard layer was mainly composed of a hard and brittle intermetallic compound, TiAl_3_, in the bonding layer. The presence of a high melting point mesophase compound that is difficult to deform may increase the hardness at the bonding layer of the Ti/Al laminated composites and reduce its bonding strength. The cracks were very easily generated in the bonding layer of the Ti/Al laminated composites during the tensile deformation process [28]. At the same time, the plastic deformation of the Ti/Al laminated composites caused its own dislocation density to increase greatly during the rolling deformation process. There were a large number of dislocations in the grains, especially near the Ti/Al interface position of the Ti/Al laminated composites [20]. As the deformation increased, dislocation entanglements caused the number of sessile dislocations to increase, which caused deformation strengthening of the composites and an increase in the hardness.

It can be seen from Figure 8 that the Ti/Al laminated composites fabricated by rolling deformation had a high hardness near the Ti/Al interface. As the distance from the Ti/Al interface increased, the hardness decreased and stabilized after reaching a certain distance. The hardness distribution may be related to an uneven force on the Ti/Al laminated composites during the rolling process. A high-speed collision at the Ti layer and Al layer would produce an extremely high pressure at the Ti/Al interface, and the metal on both sides of the interface would experience severe plastic deformation, and different degrees of work hardening would occur in the Ti layer and Al layer. Therefore, the hardness of the Ti/Al laminated composites on both sides of the Ti/Al interface increased. The closer to the interface, the greater the degree of plastic deformation was and the higher the hardness of the composites. However, away from the Ti/Al interface, the plastic deformation of Ti and Al was not obvious, and the hardness was basically consistent with that of the original base metal [19].

By comparing the microhardness in the areas near Ti/Al interface of the Ti/Al laminated composites in the 0°, 45° and 90° directions, it can be seen that the hardness in the 45° and 90° directions were basically equal, and the hardness in the 0° direction was slightly lower than that in the 45° and 90° directions. In accordance with the analysis above, grains in the Ti and Al layers in the Ti/Al laminated composites were preferentially oriented toward the rolling direction. The grains in the Ti/Al laminated composites in the 0° direction were elongated, and in the 45° and 90° directions, they were approximately equivalent and arranged in a regular fashion. It can be concluded that the microhardness along the rolling direction or in the grain elongation direction was lower than that in other directions. For the Al layer in the Ti/Al laminated composites, the microhardnesses along the 0°, 45° and 90° directions were obviously different. This may be because of the high plasticity of the Al layer, which led to a substantial plastic strain and obvious strain hardening during the rolling deformation process. For the Ti layer, the microhardnesses along the 0°, 45° and 90° directions were not significantly different. This may be because of the poor plasticity of the Ti layer, which led to a low plastic strain and lack of an obvious strain hardening during the process of rolling deformation. This is consistent with the conclusions above.

#### 3.2.2. Stress

**Figure 9:**
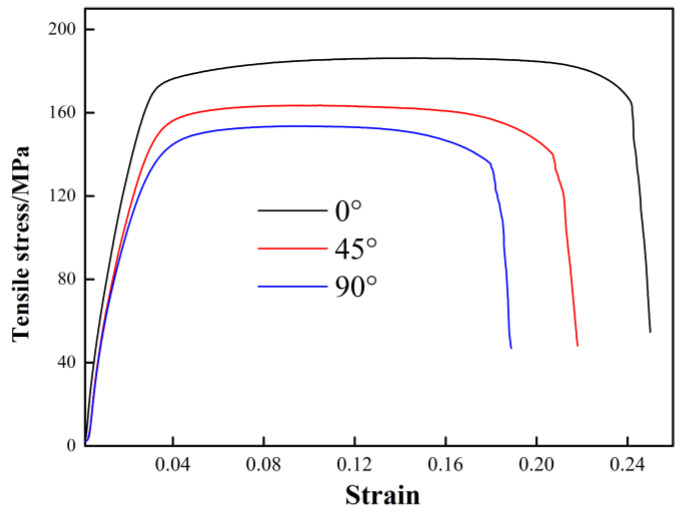
Stress-strain curves of the Ti/Al laminated composites obtained from the tensile test.

Figure 9 shows the stress-strain curves of the Ti/Al laminated composites in the 0°, 45° and 90° directions obtained from the tensile test. The gage length of the broken specimens was measured to calculate the elongation percentage of the composites, and the results are shown in Table 2. It can be seen from Figure 9 and Table 2 that the Ti/Al laminated composites fabricated by rolling in this paper had the highest elongation in the 0° direction or the rolling direction and was 24.67%. The 45° direction had the second highest elongation value of 22.25%. The elongation in the 90° direction or perpendicular to the rolling direction was the lowest and had a value of 19.18%. As shown in Figure 8, the microhardness near the Ti/Al interface of the Ti/Al laminated composites in the 90° direction was the highest herein. In accordance with the previous conclusions, the higher the hardness at the interface position was, the lower the bonding strength of the composites. During the tensile deformation, the cracks were very likely to occur in the bonding layer, and delamination cracking and damage of the Ti/Al laminated composites occurred very quickly. Therefore, the Ti/Al laminated composites in the 90° direction had a worse deformation ability and elongation than those in the 0° and 45° directions. It can be seen from Figure 9 that in the elastic deformation stage of the composites, the elastic modulus value of the specimens in the different directions were not obviously different, and the elastic deformation was not obviously correlated with the orientation. In the plastic deformation stage of the composites, the mechanical properties of the specimens in the different directions varied greatly. The tensile strength of the composites in the 0° direction was the highest and reached 186.31 MPa. The tensile strength in the 45° direction was the second highest and reached 163.07 MPa. The tensile strength in the 90° direction was the lowest and reached 154.33 MPa. The difference in the elongation of the composites was also relatively large, and it was obviously correlated with the orientation, as shown in Table 2.

#### 3.2.3. Peel Strength

**Figure 10:**
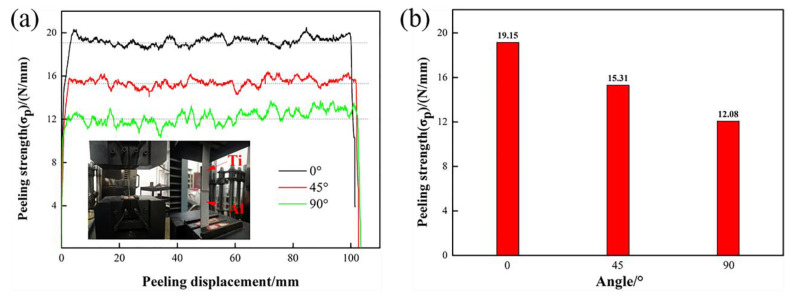
The results of the peel test. (**a**) peeling strength-displacement curves of the composites, (**b**) peeling strength with different angle of the composites.

Figure 10 shows the results of the peel test of the Ti/Al laminated composites. The average peeling strength is equal to the ratio of the average load to the width of the sheets, and the calculation results are shown in Figure 10b.

Figure 10 shows that the average peeling strength of the composites in the 0° direction was 19.15 N/mm, which is significantly higher than that in the 45° and 90° directions, which were 15.31 N/mm and 12.08 N/mm, respectively. In accordance with the results of the peel test, it can be seen that the Ti/Al laminated composites in the 90° direction readily delaminated along the interface more so than along the other directions during the uniaxial stretching process. Premature delamination means that the crack was very likely to initiate and expand in the bonding layer of the composites in the 90° direction first. Thus, a decreased plasticity and elongation of the Ti/Al laminated composites resulted. This was also discussed above.

**Figure 11:**
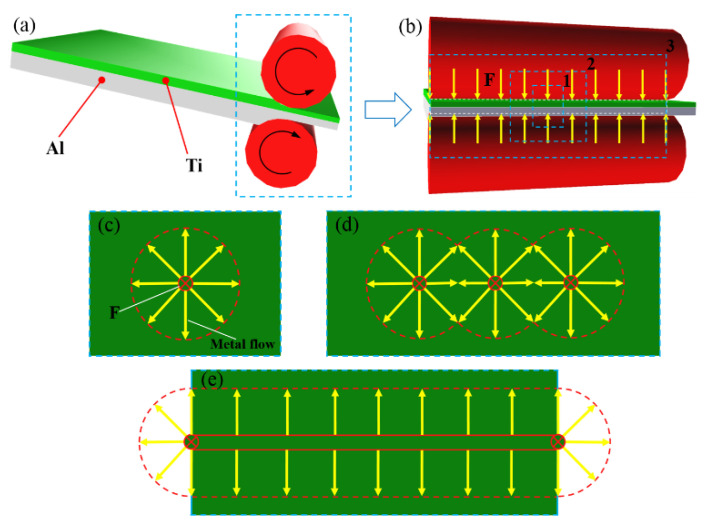
Metal flow schematic diagram of the Ti/Al laminated composites during rolling process. (**a**) rolling process, (**b**) stress distribution on the surface of the metal sheets, (**c**) metal flow at frame line 1 (single-point compression) in Figure 11b, (**d**) metal flow at frame line 2 (three-point compression) in Figure 11b, (**e**) metal flow at frame line 3 (line compression) in Figure 11b.

According to the volume conservation law, metal has a tendency to flow into the other directions that are perpendicular to the force direction when a metal is subjected to a uniaxial compressive stress. Metal flow occurs when the compressive stress is greater than the elastic deformation limit stress of the material. Figure 11 shows a metal flow schematic diagram for the Ti/Al laminated composites under a uniaxial compressive stress. For the Ti/Al laminated composites prepared in this paper, the metal flow in the width direction was hindered due to the restraining action of the rolls during the rolling deformation process, and the degree of plastic deformation was not as large as that in the rolling direction and 45° direction. In accordance with the conclusions reached by Zohreh et al. [8], the greater the rolling reduction rate is, that is, the greater the plastic deformation of the metals is, the larger the effective bonding area in the interface and the higher the peeling strength of the laminated composites. Therefore, the peeling strength of the Ti/Al laminated composites had the following order: the peeling strength in the 0° direction was the highest and reached 19.15 N/mm; the peeling strength in the 45° direction was the second highest and reached 15.31 N/mm; and the peeling strength in the 90° direction was the smallest and reached 12.08 N/mm.

### 3.3. Analysis of the Fractures of the Tensile Test Specimens

**Figure 12:**
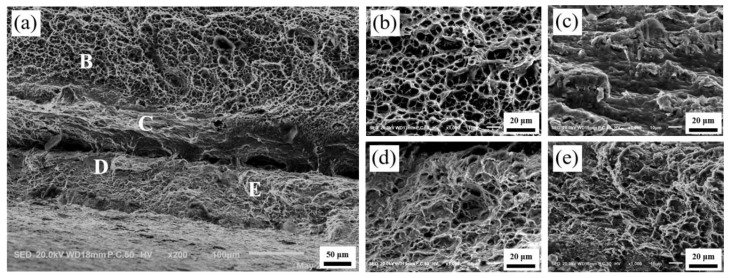
Fractography of the Ti/Al laminated composites in 0° direction after the tensile test. (**a**) Macro-fractography, (**b**) high-resolution fractography (HRF) at position B in Figure 12a, (**c**) HRF at position C in Figure 12a, (**d**) HRF at position D in Figure 12a, (**e**) HRF at position E in Figure 12a.

**Figure 13:**
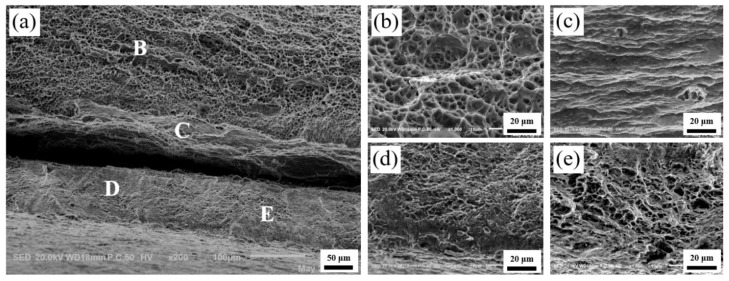
Fractography in 45° direction. (**a**) Macro-fractography, (**b**) HRF at position B in Figure 13a, (**c**) HRF at position C in Figure 13a, (**d**) HRF at position D in Figure 13a, (**e**) HRF at position E in Figure 13a.

**Figure 14:**
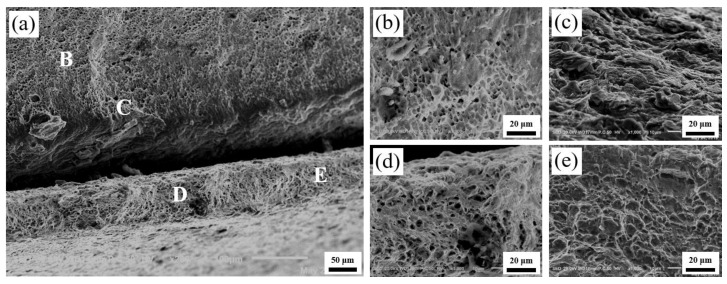
Fractography in 90° direction. (**a**) Macro-fractography, (**b**) HRF at position B in Figure 14a, (**c**) HRF at position C in Figure 14a, (**d**) HRF at position D in Figure 14a, (**e**) HRF at position E in Figure 14a.

Figure 12, Figure 13 and Figure 14 show the microscopic morphology of the fractures of the Ti/Al laminated composites in the 0°, 45° and 90° directions, respectively. Among them, the high-magnification images at positions B, C, D, and E in Figure 12a are shown in Figure 12b–e. As shown in Figure 12a, the delamination at the Ti/Al interface of the composites in the 0° direction was not obvious; that is, the bonding interface of the composites was good in this direction. The crack initiated slowly and expanded in the bonding layer of the composites during the tensile deformation process; the high plasticity of the Al and high strength of the Ti were then fully utilized [28]. At the same time, the deformation of the Ti layer and the Al layer of the composites was coordinated due to the existence of the interface, and the fracture failure of the composites was delayed, so the fracture of the Ti layer and the Al layer of the Ti/Al laminated composites was an obvious ductile fracture [31].

By observing the fracture surface of the Al layer of the Ti/Al laminated composites in the 0°, 45° and 90° directions, it can be seen that the voids in Figure 12b were small and deep, and the distribution was uniform. The voids in Figure 13b were large and shallow, and the distribution was relatively uniform. In addition to the large and shallow voids, there were large brittle sections in Figure 14b. By observing the fracture surfaces of the Ti layers of the Ti/Al laminated composites in the 0°, 45° and 90° directions, it can be seen that the large voids and brittle fractures coexisted, and the fracture morphology of the Ti/Al laminated composites in the different directions was not substantially different. By observing the bonding layer of the Ti/Al laminated composites in the 0°, 45°, and 90° directions, it can be seen that layered fractures with sharp edges appeared and indicated a typical brittle fracture morphology.

The crack first initiated in the bonding layer of the composites during the tensile deformation process. As the degree of deformation increased, the microcracks expanded and linked gradually [32]. After extending to a certain extent, the delamination occurred at the bonding interface of the composites. As shown in Figure 13a and Figure 14a, the delamination of the composites at the bonding interface can be clearly observed. By observing the fracture surfaces of the Al layer and the Ti layer, it can be seen that there were many small, deep and evenly arranged pits in the Al layer and some large and shallow pits in the Ti layer. This appearance may be because there were more or less initial microvoids in the materials [33]. When the elastic deformation energy of the materials overcame the peeling strength between the impurity elements in the Ti (Al) and the base metal, the minute voids were formed. Then, the repulsive force of the dislocations was greatly reduced, and a large number of dislocations were transferred to the new microvoids. The microvoids grew and linked gradually, and the pits formed eventually. The aggregation and growth of the microvoids were the main factors that caused the metal material fracture, and the size, number and depth of the pits were mainly determined by the toughness of the materials.

## 4. Conclusions

In this paper, the influence of the anisotropy on the microstructure and mechanical properties of Ti/Al laminated composites fabricated by rolling was studied. The following conclusions can be drawn from this study:

(1) The grains in the Al layer of the composites were elongated along the rolling direction and were compressed perpendicular to the rolling direction. The grains in the Ti layer had no obvious preferential orientation and were mainly twins. The Ti/Al laminated composites had a straight Ti/Al interface, and the interface had a small amount of waviness.

(2) The hardness values of the Al layer and Ti layer were 42 HV and 148 HV, respectively. The hardness near the bonding layer increased with increasing angle of the composites.

(3) The tensile strength, elongation and bond strength of the Ti/Al laminated composites all exhibited a significant anisotropy, and they all decreased as the angle of the composites increased.

## Figures and Tables

**Table 1:** The phase near the interface of the Ti/Al laminated composites.

Area	Ti (At%)	Al (At%)	Phase
1	100	0	Ti Matrix
2	99.98	0.02	Ti Matrix
3	98.89	1.11	Ti Matrix
4	28.32	71.68	TiAl_3_
5	0.47	99.53	Al Matrix
6	0.01	99.99	Al Matrix
7	0	100	Al Matrix

**Table 2:** Mechanical properties of the Ti/Al laminated composites.

Specimen	0°-1	0°-2	0°-3	45°-1	45°-2	45°-3	90°-1	90°-2	90°-3
Rm/MPa	186.23	189.08	183.63	163.46	162.69	163.07	152.85	156.67	153.46
σh/%	25.00	24.20	24.80	21.80	22.46	22.48	18.86	19.79	18.88
